# Porous Geometry Guided Micro-mechanical Environment Within Scaffolds for Cell Mechanobiology Study in Bone Tissue Engineering

**DOI:** 10.3389/fbioe.2021.736489

**Published:** 2021-09-14

**Authors:** Feihu Zhao, Yi Xiong, Keita Ito, Bert van Rietbergen, Sandra Hofmann

**Affiliations:** ^1^ Orthopaedic Biomechanics, Department of Biomedical Engineering, Eindhoven University of Technology, Eindhoven, Netherlands; ^2^ Institute for Complex Molecular Systems (ICMS), Eindhoven University of Technology, Eindhoven, Netherlands; ^3^ Zienkiewicz Centre for Computational Engineering, Faculty of Science and Engineering, Swansea University, Swansea, United Kingdom; ^4^ School of System Design and Intelligent Manufacturing, Southern University of Science and Technology, Shenzhen, China

**Keywords:** micro-mechanical environment, mechanical stimulation, scaffold porous geometry, mechanobiology, bone tissue engineering

## Abstract

Mechanobiology research is for understanding the role of mechanics in cell physiology and pathology. It will have implications for studying bone physiology and pathology and to guide the strategy for regenerating both the structural and functional features of bone. Mechanobiological studies *in vitro* apply a dynamic micro-mechanical environment to cells *via* bioreactors. Porous scaffolds are commonly used for housing the cells in a three-dimensional (3D) culturing environment. Such scaffolds usually have different pore geometries (e.g. with different pore shapes, pore dimensions and porosities). These pore geometries can affect the internal micro-mechanical environment that the cells experience when loaded in the bioreactor. Therefore, to adjust the applied micro-mechanical environment on cells, researchers can tune either the applied load and/or the design of the scaffold pore geometries. This review will provide information on how the micro-mechanical environment (e.g. fluid-induced wall shear stress and mechanical strain) is affected by various scaffold pore geometries within different bioreactors. It shall allow researchers to estimate/quantify the micro-mechanical environment according to the already known pore geometry information, or to find a suitable pore geometry according to the desirable micro-mechanical environment to be applied. Finally, as future work, artificial intelligent – assisted techniques, which can achieve an automatic design of solid porous scaffold geometry for tuning/optimising the micro-mechanical environment are suggested.

## Definition

Scaffold pore geometry that is presented in this review involves the following parameters:

• Pore shape: the architecture or shape of the scaffold micro-pores, which can be irregular or regular (cubic, spherical, gyroid, etc.);
• Pore dimension: also called pore size or pore diameter, which is a measure of the (maximal) distance between two neighbouring struts, usually has a value around 100–2000 µm for bone tissue engineering applications;
• Porosity: also called void fraction, which is a measure of the void (i.e. “empty”) spaces in scaffolds, and has a value in the range of 0–100%.

## Introduction

In the field of bone tissue engineering (BTE), a primary challenge is to recapitulate both the structural and functional features of bone (Amini et al., 2012). Mechanobiology research seeks to understand the role of mechanics in cell physiology and pathology. Bone cells are known as mechanosensitive cells that respond to their mechanical environment *in vivo* and *in vitro* (Klein-Nulend et al., 2003; Giorgi et al., 2016). Mechanobiology research in BTE aims at getting insight into how the scaffolds or the application of mechanical loads affect the development of tissue-engineered bone tissue, which is intended to be used for bone disease research, drug testing, etc. (García-Aznar et al., 2021; Kim et al., 2021). *In vitro* mechanobiology includes the creation of either static or dynamic micro-mechanical environments. The cellular mechanical environment is then transduced into biochemical signals through mechano-transduction protein networks, which therefore influence the cellular behaviours, such as osteogenic differentiation of stem cells in BTE (Delaine-Smith and Reilly, 2012; Jansen et al., 2015; Paluch et al., 2015; Wittkowske et al., 2016; Naqvi and McNamara, 2020). A static micro-mechanical environment refers to the use of biomaterials with different mechanical properties to which the cells attach. The effect of mechanical properties inherent to biomaterials on bone cell behaviour have been widely reviewed, e.g. by Janmey et al. (2020), Klein-Nulend et al. (2012), Lin et al. (2020), Selig et al. (2020) or Janmey et al. (2020), to name a few. This review will focus on the dynamic micro-mechanical environment on cells that is guided by the scaffold pore geometry when loading is applied through the use of bioreactors.

Various bioreactors are being applied in BTE. For example, flow perfusion bioreactors, spinner flasks or rotating wall vessels can be used which all apply a fluid induced wall shear stress (WSS) on cells (Granet et al., 1998; Sikavitsas et al., 2002; Bancroft et al., 2003). Mechanical compression and stretching bioreactors can be used for applying mechanical strain to cells that are attached on scaffold struts (Zhang et al., 2008; Bilgen et al., 2013). For cell culturing in 3D, scaffolds are used for housing and supporting the seeded cells. Scaffolds used in the experiments usually have different porous geometries, for example some have irregular pore shapes (Mccoy et al., 2012), and some have regular pores but with different porosities or pore dimensions (Bartnikowski et al., 2014). With improvements in 3D printing/additive manufacturing technology, scaffolds with well-defined geometries can be manufactured, and this will probably be the standard for scaffold manufacturing in the near future (Bahraminasab, 2020). To investigate the influence of scaffold pore geometry on the internal micro-mechanical environment, computational approaches are commonly used, thanks to the capability of such approaches to calculate/simulate the mechanical environment at the micro (or even sub-micro) scale with low cost, which is challenging for experimental measurements (García-Aznar et al., 2021). It has been found that the scaffolds’ pore geometry can largely influence the micro-mechanical environment within the scaffolds (Olivares et al., 2009). Previous examples are the computation of the fluid flow induced micro-mechanical environment when applying flow perfusion-, spinner flask- or rotating wall vessel bioreactors by the application of computational fluid dynamics (CFD). Or the mechanical deformation (such as stretching/compression) of the cells within scaffolds in compression/stretching bioreactors (Brunelli et al., 2017), where finite element (FE) models based on fluid-structure interaction (FSI), biphasic poro-elasticity, etc. have been used for simulating/quantifying the resultant WSS and/or mechanical strain on scaffold struts (Zhao et al., 2016; Castro and Lacroix, 2018).

This review aims at providing insight into the role of scaffold pore geometry parameters (i.e. porosity, pore dimension and pore shape) based on previous theoretical studies, in order to better understand their complex effect on the micro-mechanical environment of bone cells. It will benefit the BTE/bone organoids fields for cellular mechanobiology research. For example, this information is expected to allow researchers to estimate the micro-mechanical environment depending on scaffold geometry information, or to find/design a suitable pore geometry providing a desirable micro-mechanical environment to the cells. The limitations of the current computational approaches in automatically achieving a scaffold geometry design that is driven by micro-mechanical environment will be discussed. An outlook and suggestions for future research in terms of artificial intelligence (AI) – assisted techniques for addressing the limitations in scaffold geometry design will be presented.

## The Role of Scaffold Pore Geometry on the Cell Micro-mechanical Environment

This section will present the influence of the scaffold pore geometry, more specifically pore shape, pore dimension and porosity on the resultant WSS and mechanical strain within empty scaffolds in perfusion, spinner flask and compression bioreactors.

### Assumptions for Calculating the Cell Micro-mechanical Environment Within Scaffolds

**FIGURE 1:**
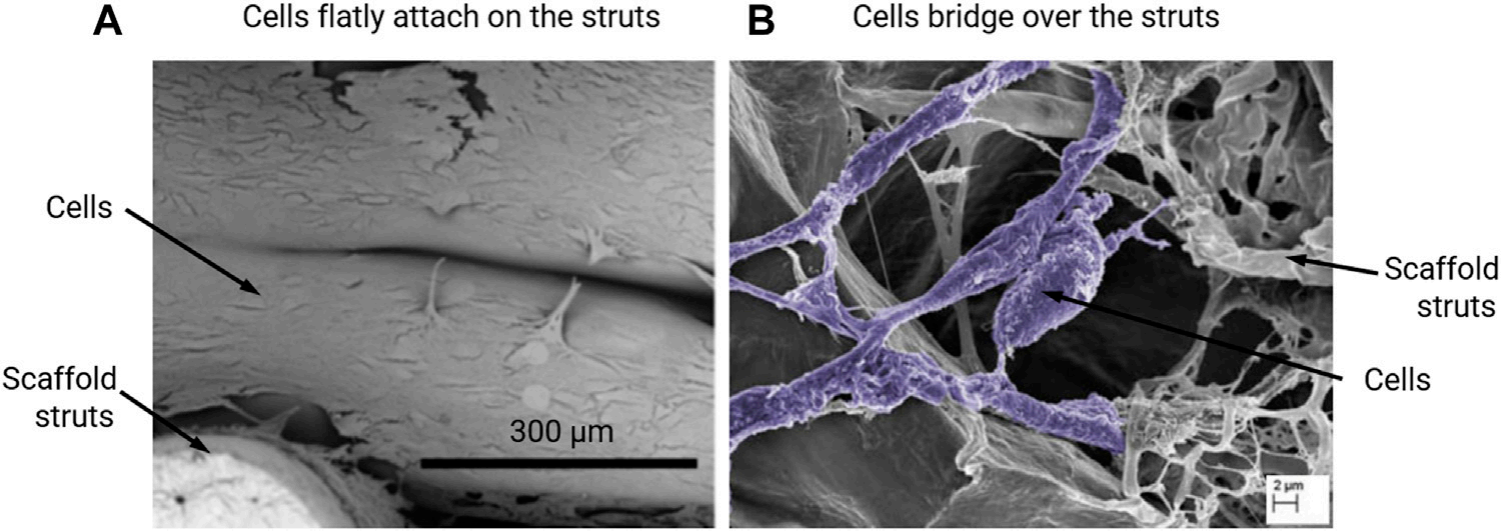
Scanning electron microscopy (SEM) images of MC3T3-E1 cells that **(A)** flatly attach on the Poly-lactic acid (PLLA) scaffold struts on day 7 of culturing, re-produced from (Xue et al., 2019); or **(B)** bridge over the collagen glycosaminoglycan (CG) scaffold struts on day 6 of culturing, re-produced from (Mccoy et al., 2012).

The calculation of fluid – induced WSS within empty scaffolds is based on the assumption that the WSS at the scaffold surface is a good representation of the WSS sensed by the cells that are attached to the scaffold surfaces. It also assumes that the cells attach mostly flat to the scaffold surface in the initial state, with a minimal cell volume with respect to the pore volume. This assumption has been shown to be met for some experiments/scaffold materials (Figure 1A), but not for all (Figure 1B). For calculating the mechanical strain in empty scaffolds, it is assumed that the cells are subjected to the strain magnitude at the location of the scaffold that they are attached to (Olivares et al., 2009; Laurent et al., 2014). Such assumptions can be reasonable if the scaffold material is much stiffer than the cell and no substantial ECM has been formed yet.

### Porosity

Porosity is the main determinant for scaffold permeability and thus the amount of flow through the scaffold in perfusion/stirring bioreactors and was found to be an important parameter determining the results of BTE (e.g. seeded cell density, cell proliferation, ECM production, etc.) (Grayson et al., 2008; Panseri et al., 2021). The effect of porosity on the permeability of the scaffold, which describes the amount of flow through the scaffold, can be calculated by the Kozeny-Carman Equation (Eq. 1) (Van Bael et al., 2012; Egan, 2019):

$$\kappa =\left(\frac{1}{C_{k}}\right)\cdot \frac{\varphi }{S_{s}^{2}}$$ (1)

where, *κ* is the permeability, *φ* is the porosity, *c*_*k*_ is the Kozeny constant and *S*_*s*_ is the specific surface area calculated as the surface area divided by the total volume of the struts.

**FIGURE 2:**
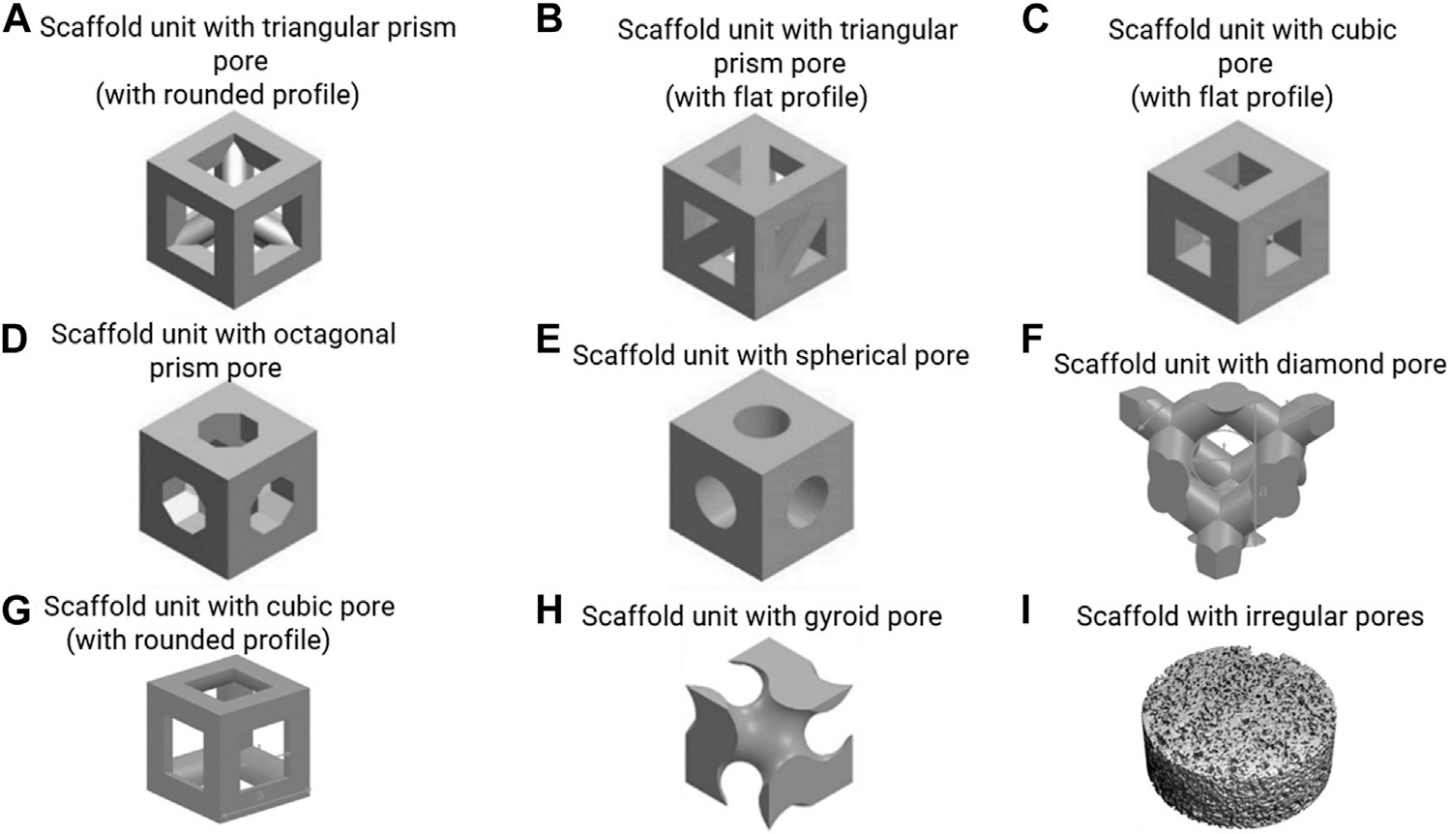
**(A**–**H)** scaffold units with various regular pore shapes, re-produced from (Lu et al., 2020; Deng et al., 2021; Prochor and Gryko, 2021); **(I)** scaffold with irregular pores, re-produced from (Zermatten et al., 2014).

This equation demonstrates that scaffold permeability linearly increases with porosity. This has also been demonstrated by experimental measurements (Zhang et al., 2019). The relationship between the permeability, the fluid velocity and the WSS, however, is complex and also depends on the pore geometry. Ali and Sen (2018) employed a CFD approach to investigate the influence of porosity on the permeability and WSS, and they found that under a fluid velocity of 0.7 mm/s, the average WSS decreased from 131 to 27 mPa with an increase in porosity from 65 to 90% for the gyroid pore shape (Figure 2H). This trend also happened for a diamond pore shape (Figure 2F) (Ali and Sen, 2018). Melchels et al. (2011) designed and manufactured a scaffold (gyroid pores in Figure 2H) with different porosities (40–85%) in different regions. This resulted in different shear rates (SRs) in the regions with different porosities under perfusion flow, e.g. 10–40 s^−1^, i.e. higher SR in the region with higher porosity (Melchels et al., 2011).

Porosity is also the main determinant for the amount of strain in the scaffold when an external force is applied according to both micro-FE analyses and experimental characterisation (Hannink and Arts, 2011; Castro et al., 2020). If under pressure/compressive force loading through bioreactors, a scaffold with a lower porosity (i.e. higher overall structural stiffness) will show less deformation, thus cells that attach on the struts will receive lower strain. Not only for overall structural stiffness, the porosity also can influence the local stress/strain concentration under compression loading. For instance, in the computational study by Hendrikson et al. (2017), scaffolds that had a cubic pore shape, a pore dimension of 151 μm, but different porosities (74 vs 64%) were compared in terms of octahedral shear strain under a compressive strain of 10%. It was found that the scaffold with higher porosity (74%) had a larger strut area in the low strain range (e.g. <10%) than the one with lower porosity (64%) (Hendrikson et al., 2017). Similarly to section 2.2 and 2.3, under dynamic compression, the porosity will also have an influence on the resultant WSS that is induced by fluid flow due to compression. Zhao et al. (2016) compared scaffolds with porosities of 60–90% and found that a higher porosity resulted in lower WSS under dynamic compression. By increasing the pore dimension, the influence of porosity on WSS became smaller, e.g. for cubic pore, dimension = 100 µm: average WSS = 3.5 mPa when porosity = 60%, average WSS = 2.5 mPa when porosity = 90%; for cubic pore, dimension = 300 µm: average WSS = 1.5 mPa when porosity = 60%, average WSS = 1.1 mPa when porosity = 90% under dynamic compression (strain = 1%, frequency = 1 Hz) (Zhao et al., 2016). So, under dynamic compression, the lower porosity and pore dimension can result in higher fluid flow-induced WSS.

### Pore Dimension

Pore dimension is the main factor that determines fluid-induced WSS under perfusion flow (Fu et al., 2021). Also, pore dimension is one of the factors that can influence cell attachment (e.g. flatly attached on struts/bridging over struts in Figure 1) (Guo et al., 2015; Yamashita et al., 2016). Previous mechanobiological studies have investigated cell responses via tuning the scaffold pore dimensions while keeping the pore shape constant (Bartnikowski et al., 2014; Ouyang et al., 2019). For example, Bartnikowski et al. (2014) quantified the WSS within the scaffolds that had cubic pores (with rounded profile in Figure 2G) and a porosity of 60% but with different pore dimensions (625 vs 1250 µm). It was found that the scaffold with smaller pore dimension provided a higher WSS: maximum WSS = 1979 mPa/average WSS = 500 mPa (pore dimension = 625 µm) vs maximum WSS = 837 mPa/average WSS = 120 mPa (pore dimension = 1250 µm) under a flow rate of 1 ml/h (0.61 µm/s). These scaffolds were then applied in an *in vitro* cell experiment where it was found that the DNA amount was significantly higher in the cell seeded scaffold with larger pore dimension (1250 µm) (Bartnikowski et al., 2014). Whereas the accurate calculation of the actual WSS requires performing a CFD analysis for the (often complex) scaffold pore geometries, simple mathematical equation can be used to estimate the WSS (Zhao et al., 2016):

$$\frac{\tau_{a}}{v}=a_{1}\cdot {\left(\frac{d}{L_{c}}\right)}^{b_{1}}$$ (2)

Where, *τ*_*a*_ is the average WSS within the scaffold, *v* the applied fluid velocity, *d* the pore dimension, *L*_*c*_ is the characteristic length (*L*_*c*_ = 1 µm), and *a*_*1*_ and *b*_*1*_ constants that depend on the pore shape and porosity. A limitation of this approach is that the equations are only a good approximation for a limited set of pore shapes (i.e. cube with flat profile and sphere in Figures 2C,E) and porosities (i.e. 60%–90%).

Dynamic compression not only generates mechanical strains in the struts but can also result in WSS on the strut surfaces, which was mostly ignored in previous mechanobiological studies regardless of the compressive strain magnitude or frequency. It was found that the resultant average WSS was proportional to the applied compressive strain (Milan et al., 2009). Moreover, it was highly dependent on the scaffold pore dimensions (Zhao et al., 2016). Here also, simple equations were introduced to estimate the WSS due to compression (Zhao et al. (2016)):

$$\frac{\tau_{a}}{\varepsilon_{app}}=a_{2}\cdot {\left(\frac{d}{L_{c}}\right)}^{b_{2}}$$ (3)

where, *τ*_*a*_ is the average WSS within the scaffold, *ε*_*app*_ is the applied compressive strain by bioreactor, *d* the pore dimension, *L*_*c*_ is the characteristic length (*L*_*c*_ = 1 µm), and *a*_*2*_ and *b*_*2*_ constants that depend on the pore shape and porosity. Similar as in Eq. 2, one of the limitations of this approach is that the equations are a good approximation only for a limited set of pore shapes (i.e. cube with flat profile and sphere in Figures 2C,E) and porosities (i.e. 60%–90%). Also, this Eq. 3 is only applicable for a dynamic compression frequency of 1.0 Hz and needs to be adapted to other frequencies. For all other cases beyond the aforementioned ones, a FSI analyses will be needed to accurately calculate the WSS.

Under mechanical compression, the stress/strain distribution can be influenced by the pore dimension. Ribeiro et al. (2017) investigated this based on the scaffolds with pore dimensions of 740 and 370 µm using an FE model. There, an unconfined compression loading with a strain of 15% was applied on both scaffolds. Their results showed that the maximum value of compressive stress was similar between the two scaffolds with different pore dimensions, i.e. maximum compressive stress = 27.7 MPa in pore dimension of 740 µm vs maximum compressive stress = 25.9 MPa in pore dimension of 370 µm. However, the scaffold with larger pores (pore dimension = 740 µm) had more regions (area) with higher stress than the one with smaller pores (pore dimension = 370 µm) (Panadero et al., 2015).

### Pore Shape

The effect of pore shape on the fluid-induced WSS in flow perfusion and spinner flask bioreactors is difficult to predict. Some scaffolds with different regular pore shapes (but same pore size and porosity) have similar WSS, but some do not. The commonly (designed) regular pore shapes include sphere, cube, gyroid, prism, etc. (Figures 2A–H), which can be manufactured by 3D printing/additive manufacturing technology. Prochor and Gryko (2021) quantified the WSS within scaffolds that have different regular pore shapes (e.g. triangular prism with rounded and flat profiles, cube, octagonal prism and sphere in Figures 2A–E) under perfusion flow. It was found that the scaffold with triangular prism (with rounded profile) experienced the highest WSS, whereas the scaffold with spherical pores experienced the lowest WSS under the same flow rate. The maximum WSS within spherical pores and cubical pores were identical. However, this can be different within different bioreactors that generate fluid – induced WSS. In a combined experimental and computational BTE study by Rubert et al. (2020), the average WSS within the scaffolds with spherical pores (average pore diameter = 330 μm, porosity = 84.7%) and cubical pores (average pore diameter = 330 μm, porosity = 92.8%) were 0.42 and 0.81 mPa respectively in a spinner flask (70 RPM). This was associated to upregulated osteoblast cell differentiation and ECM formation within cubic pores, while ECM mineralisation was enhanced within the spherical pores (Rubert et al., 2020).

Porous scaffolds also can have irregular pore shapes, which are for example obtained from more traditional fabrication methods such as porogen leaching (Figure 2I). Studies have found that the irregularity of the pore shape does not have a distinct influence on the fluid – induced WSS, once the pore dimensions and porosity are similar. For example, Zermatten et al. (2014) investigated the influence of the pore irregularity on the internal WSS using scaffolds with regular cubical pores (with rounded profile) and highly irregular pores (Figures 2G,I). Although the other two parameters, pore dimensions (regular: 0.22 mm vs irregular: 0.16 mm) and porosity (regular: 38% vs irregular: 55%) were not exactly the same, the average WSS within irregular and irregular pores have high similarity (regular: 3.08 mPa vs irregular: 3.68 mPa) under a perfusion fluid velocity of 0.066 mm/s (Zermatten et al., 2014). One limitation of simulating the micro-fluidic environment within these highly irregular pores (at whole scaffold level) was the high computational cost (Santamaría et al., 2013; Zermatten et al., 2014). To address this challenge, Zhao et al. (2019) developed a more versatile technique, creating a multiscale and multiphasic CFD model. In this approach, small but representative parts of the scaffold are being used for the generation of a microstructural model of the pore environment, which are then coupled with a macro-model representing the whole scaffold in which the microstructure is homogenised. The macro-model can be used to calculate the fluid flow at larger length scales, that then can be applied to the micro-model to calculate the local WSS at the cell level. As only small parts of the scaffold need to be modelled in detail, this approach can reduce the computational costs while still providing results at the cell-level. It has been shown that with this multiscale and multiphasic CFD model, calculations of resulting WSS within any scaffold with highly irregular pore shape is possible even using a normal computer (e.g. 16 GB RAM, Intel i7 CPU). However, this technique requires that the Reynolds (Re) number should less than 1 when using Darcy’s law for homogenisation (Chaudhary et al., 2011).

Under mechanical compression, the pore shape can have a distinct influence on the overall structural stiffness of the scaffold (Castro et al., 2020; Jahir-Hussain et al., 2021). According to their calculations, the triangular pore shape resulted in the highest structural stiffness of the scaffold and the spherical pore shape resulted in the lowest stiffness among the various pore shapes (spherical, cubic, hexagonal and triangular). Under pressure/compressive force, scaffolds with a lower structural stiffness (e.g. with spherical pores) will have a higher strain in the struts than the ones with a higher structural stiffness (e.g. with cubic/hexagonal/triangular pores). This difference will translate to differences in strain sensed by cells attached to the struts. In cell culture experiments applying compression to stimulate cells, usually dynamic compression is applied. As mentioned in sections 2.2 and 2.3, this dynamic compression also generates WSS within the pores of the scaffold. A FSI approach for quantifying the WSS during dynamic compression has found that the WSS was higher within spherical pores than that within cubical pores, e.g. 5.5 mPa within spherical pores (pore diameter = 100 μm, porosity = 60%) and 3.5 mPa within cubical pores (pore diameter = 100 μm, porosity = 60%) under an applied compressive strain of 1.0% and at a frequency of 1.0 Hz (Zhao et al., 2016). Therefore, to precisely quantify the WSS due to dynamic compression, the pore shape needs to be explicitly reflected in the computational model.

Some scaffolds have extremely anisotropic pores, such as those with unidirectional channels or holes, as shown in (Deville et al., 2006, 2007; Munch et al., 2009; Pourhaghgouy et al., 2016). For this type of scaffolds, the above discussed influence of porosity, pore dimension and pore shape on the internal micro-mechanical environment is still applicable for external loading in the unidirectional orientation (e.g. fluid perfusion/unidirectional mechanical compression/stretching), but not for external loading in non-unidirectional directions (e.g. spinner flask/non-unidirectional compression/stretching).

## Effect of Cell/tissue Growth on the Micro-mechanical Environment Within Scaffold Pores

A major limitation of all studies above is that they do neither consider the cells nor the tissue within the scaffold pores. In these studies, it is assumed that the cells lie flatly attached to the scaffold surface and that their volume is small compared to the pore volume. In other situations, e.g. when cells can bridge across the pores (Figure 1B) (Mccoy et al., 2012), this assumption no longer holds and can lead to large errors when calculating the WSS. Moreover, once tissue starts to form within the scaffold, its porosity, and consequently the micro-mechanical environment, can dramatically change (Sandino and Lacroix, 2011). The influence of scaffold pore geometry on the micro-mechanical environment when considering cell/tissue growth has not been as rigorously investigated as within empty scaffolds. In this section, some computational models which can simulate the micro-mechanical environment while considering cell/tissue within scaffolds are reviewed.

### Cells Within Scaffold Pores

In some BTE experiments, dynamic cell seeding is used for improving the seeding efficiency and/or distribution of seeded cell in the porous scaffolds. Perfusion flow is usually used for dynamic seeding. During this process, the fluid force can also mechanically stimulate the cells through cell deformation which consequently can promote cellular processes (Rüberg and Aznar, 2016; Serrano-Alcalde et al., 2017).

**FIGURE 3:**
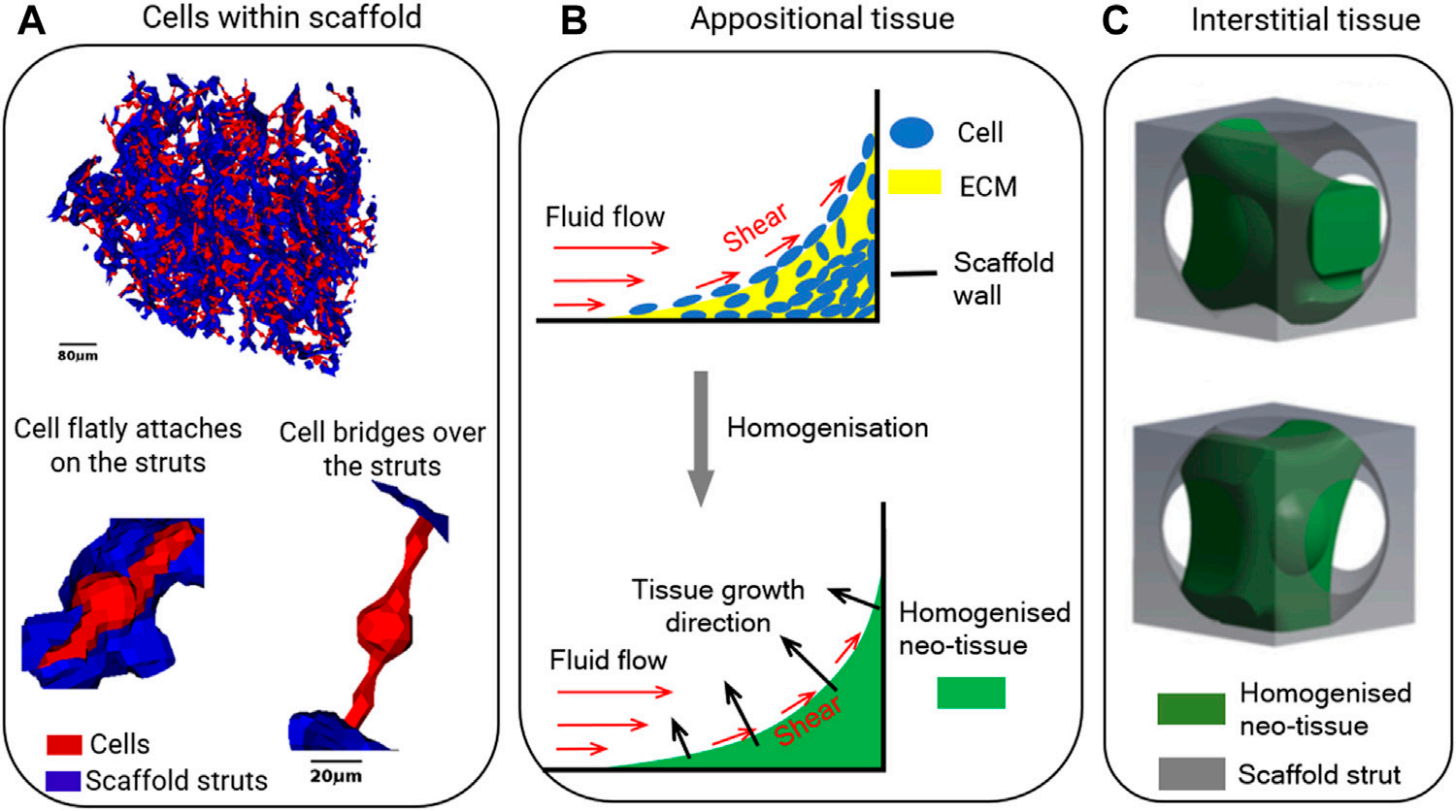
Illustrations of **(A)** cells within the scaffold in computational model, re-produced from (Jungreuthmayer et al., 2009); **(B)** appositional tissue growth in computational model, re-produced from (Zhao et al., 2020a); **(C)** interstitial tissue within unit scaffold in computational model, re-produced from (Zhao et al., 2020b).

For seeded cells, Jungreuthmayer et al. (2009) and Mccoy et al. (2012) modelled cells as flatly attached and as bridged morphologies within collagen glycosaminoglycan (CG) scaffolds, which had irregular pore shapes (Figure 3A). It was found that the influence of cell morphology (attached/bridged) on the cellular WSS depends on the locations within scaffolds (Guyot et al., 2016b). Furthermore, in the study by Mccoy et al. (2012), three CG scaffolds with different pore dimensions (85, 120 and 325 µm) but equal porosity (90%) were compared in terms of resultant WSS on cells. It was found that the average WSS on all cells (both bridged and attached morphologies) was 165, 176 and 155 mPa, respectively for the pore dimensions of 85, 120 and 325 µm under a perfusion fluid velocity of 235 µm/s, and the WSS was proportional to the fluid velocity (Mccoy et al., 2012).

### Tissue Growth Within Scaffold Pores

To investigate the influence of scaffold pore geometry on the changing micro-mechanical environment over time, tissue growth models have been introduced. These are coupled with FE/CFD models. To account for tissue growth, various studies have developed mathematical models to describe neo-tissue formation assuming appositional growth in static conditions (Nava et al., 2013; Guyot et al., 2014; Herklotz et al., 2015; Egan et al., 2018). In the mathematical model, the cells and ECM are usually homogenised (Figure 3B). One of the most commonly used models for describing the tissue growth kinetics is based on the level set (LS) method, which is available in both commercial and open-source software packages (e.g. COMSOL, ANSYS, OPENFOAM, etc.). It allows to model appositional tissue growth as illustrated in Figure 3B. The governing equation is (Guyot et al., 2014):

$$\frac{\partial \psi }{\partial t}+\left(V_{G}\cdot n_{\Gamma }\right)\cdot \nabla \psi =0$$ (4)

where, **n**_**Γ**_ is the normal unit vector to the interface between neo-tissue and medium domains, *ψ* is the LS function and *V*_*G*_ is neo-tissue growth velocity governed by local the struts curvature *κ*_*c*_ in Eq. 5:

$$V_{G}=\{\begin{array}{c} -\lambda \cdot \kappa_{c}\;\;\;\;\;\left(\kappa_{c}>0\right)\\ 0\;\;\;\;\;\;\;\;\;\;\;\;\;\;\;\left(\kappa_{c}\leq 0\right) \end{array}$$ (5)

Where, *λ* is the tissue growth rate.

To investigate the influence of the micro-mechanical environment changes during neo-tissue growth within scaffolds that have different pore geometries, the tissue growth model needs to be coupled with the FE/CFD model by introducing a WSS-dependent term into Eq. 5. The WSS (*τ*) in Eqs 6, 7 by Guyot et al. (2015, 2016a) then is computed by a CFD model:

$$\;V_{G}=\{\;\begin{array}{c} -A\cdot \kappa \cdot f\left(\tau \right)\;\;\;\;\;\kappa \;>\;0\\ 0\;\;\;\;\;\;\;\;\;\;\;\;\;\;\;\;\;\;\;\;\;\;\;\;\kappa \;\leq \;0 \end{array}$$ (6)

$$f\left(\tau \right)=\{\begin{array}{c} 0.5+\frac{0.5\tau }{a_{1}}\;\;0\leq \tau \leq a_{1}\\ 1\;\;\;\;\;\;\;\;\;\;\;\;\;\;\;\;\;\;\;a_{1}\leq \tau \leq a_{2}\\ \begin{array}{c} \frac{\tau -a_{3}}{\;\;a_{2}-\;\;a_{3}}\;\;\;a_{2}\leq \tau \leq a_{3}\;\;\;\\ \;0\;\;\;\;\;\;\;\;\;\;\;\;\;\;\;\;\;\tau \geq a_{3} \end{array} \end{array}$$ (7)

Where, *a*_*1*_ and *a*_*2*_ are the minimal and maximal shear stresses enhancing neo-tissue formation and *a*_*3*_ the critical shear stress.

Then the computational model is applied to scaffolds that have different pore geometries. For instance, Guyot et al. (2015) applied the model on scaffolds with two different pore shapes (i.e. cubic shape with rounded profile and pore dimensions of 650 µm vs diamond shape and pore dimension of 750 µm in Figures 2F,G). It was found that under the same amount of tissue produced within the scaffolds higher shear stress occurred in the neo-tissue within the scaffold with diamond shape than that in the scaffold with cubic shape, e.g. when 30% of the porous volume was filled with neo-tissue, the average shear stress in the neo-tissue was 175 mPa within the diamond pores, while it was 80 mPa within the cubic pores (Guyot et al., 2015).

One of the limitations of these computational models is the uncertainty of the parameter values (such as *λ*, *a*_1_, *a*_2_ and *a*_3_ in Eqs 5–7). As these are empirically determined constants, they may need to be changed depending on parameters that influence tissue formation (e.g. the number of cells in the culture, the type of cells, scaffold-related attachment of cells, the density of the deposited ECM and whether or not it is mineralised). Whereas after fitting these constants to experimental results these equations thus may well describe the effect of changes made within that specific experiment, they may not well describe the outcome of other experiments. To reduce the number of parameters in the tissue growth model, recent computational studies have employed second order diffusion equations to model tissue growth kinetics (Buenzli et al., 2020; Zhao et al., 2020a). The main advantage of using this diffusion equations over the LS method is that fewer parameters need to be determined. For example, diffusion equations can already model the curvature – dependent tissue growth without adding the curvature parameter *κ* in the equation as that in LS method (Buenzli et al., 2020). Therefore, in modelling the scaffold pore geometry for tissue growth kinetics, if the curvature is not a parameter that needs to be explicitly assessed, a computational model based on a diffusion equation will be a good choice. Otherwise, a computational model based on LS method is suggested. Another limitation is that these computational models assume appositional tissue formation towards the centre of the pores. In reality, however, interstitial formation, in which the tissue is infiltrated within the pores rather than being attached on the struts surfaces is also observed in many cases (Li et al., 2009) (as illustrated in Figure 3C). The resultant WSS on cells under interstitial tissue formation was quantified and compared to appositional tissue formation (Zhao et al., 2020b). Distinct difference in WSS between two cases were found, even if the same amount of newly formed tissue was present. This implies that computational models that assume appositional tissue growth cannot well predict the micro-mechanical environment in case of substantial interstitial tissue formation. Estimating the influence of scaffold pore geometry on the micro-mechanical environment by taking the tissue into account also needs to consider whether the cell/tissue growth is appositional or interstitial. Even then, this remains challenging due to the high variability in tissue formation.

Different from LS method and diffusion equation, some other computational studies employed a simple voxel – FE based method to simulate the tissue growth within scaffolds (Adachi et al., 2006; Nasello et al., 2021). In this method, modelling the neo-tissue generation within scaffolds was achieved by adding elements on the scaffold surfaces according to the applied stress in elements where the cells are located. Therefore, this voxel – FE based method does not require mathematical functions for tissue growth kinetics. However, this method is limited to simulate the neo-tissue growth under mechanical stimulation only, and not under static conditions.

## Outlook

This review provides an insight on how scaffold pore geometry influences the micro-mechanical environment within scaffold pores, i.e. the environment that cells are subjected to. This information would allow researchers to estimate/quantify the micro-mechanical environment according to the already known pore geometry information, or to find a suitable pore geometry according to the desirable micro-mechanical environment to be applied. It also indicates which computational technique could be used for modelling the scaffold in each specific circumstance (e.g. under perfusion flow/spinner flask/compression). So far, these investigations are still in their infancy, in which a large number of scaffold geometries need to be computationally modelled, from which then the users can select suitable ones. A truly automatic optimisation of the scaffold design would obviously involve a much more rigorous approach involving search algorithms. Considering the large number of variables involved, the complexity of the design space, and the time-dependent behaviour of the problem, classical optimisation procedures are not well suited for this task. New techniques, such as an AI-assisted design pipeline centred around the computational methods/tools) could be used for addressing these limitations. To establish an AI-assisted design pipeline, several steps are needed. First, a generative computer-aided design method that can model both periodic and stochastic scaffolds will be needed to greatly enlarge the design space (Tang et al., 2020). These scaffolds with complex biomimetic designs may possess enormous potential to advance the performance of mimicking the *in vivo* condition. Second, model order reduction methods, which have been used for designing additive manufacturing products (e.g. by Xiong et al. (2019)) are needed to speed up the computer simulations, such that large training sets become available. Third, a systematic method to determine the relationship between multiple factors (e.g. scaffold geometry parameters, mechanical properties of scaffold material, chemical composition, cell attachment sites etc.) during the experimental cell mechanobiology study are needed for developing an AI-assisted design pipeline. To do this, we suggest a combination of experimental methods (e.g. adaptive sampling) and a data-driven modelling approach, which will enable the application of more advanced tasks, such as multi-task/purpose and active learning. After training, it then would be possible to suggest an optimal scaffold for a specified set of requirements with no or minimal additional computational analyses.
